# Can prolonged P-R interval predict clinical outcomes in non-ST elevation acute coronary syndrome patients?

**DOI:** 10.1186/s12872-024-03809-y

**Published:** 2024-03-02

**Authors:** Mohammad Zareei, Hossein Zareiamand, Mahsa Kamali, Nasim Ardalani, Ata Ebrahimi, Maryam Nabati

**Affiliations:** 1 Faculty of Medicine, Sari Branch, Islamic Azad University, Sari, Iran; 2 Department of Cardiology, Faculty of medicine, Islamic Azad University, Sari branch, Sari, Iran; 3 Cardiovascular Research Center, Mazandaran University of Medical Sciences, Sari, Iran; 4 Department of Cardiology, Faculty of Medicine, Cardiovascular Research Center, Mazandaran University of Medical Sciences, Sari, Iran

**Keywords:** P-R interval, ST-elevation, Electrocardiography, Coronary angiography, Echocardiography, Clinical outcome

## Abstract

**Background:**

The present study aimed to respond to clinical question, can prolonged P-R interval predict clinical outcomes in non-ST elevation acute coronary syndrome patients?

**Methods:**

This descriptive-analytical study was conducted on cardiac patients. All of the non-ST elevation acute coronary syndrome (NSTEACS) including non-ST elevation myocardial infarction (NSTEMI) and unstable angina patients included in the study. Then they divided into two groups: prolonged P-R interval and normal P-R interval. The patients who had a history of digoxin and calcium channel blocker use, using antiarrhythmic drugs, known valvular or congenital heart disease and connective tissue, unreadable P-R interval and cardiac block were excluded. Data were collected using the questionnaire consisted demographic data and clinical outcomes and a follow-up part was completed by one of the researchers.

**Results:**

Finally, 248 patients completed the study. The results showed both of the two groups had significant differences in terms of the history of myocardial infarction (MI) (*p* = 0.018), the level of high-density lipoprotein (HDL) (*p* = 0.004), heart rate (*p* = 0.042), inverted T wave (*p* = 0.017), anterior ST- segment depression (*p* = 0.008), normal report of coronary angiography (CAG) (*p* = 0.003), three vessels disease (*p* = 0.043), left main lesion (*p* = 0.045) and SYNTAX score (*p* = 0.032) based on the CAG report. The results of six-month follow-up showed although, the frequency of ischemic stroke, coronary artery disease (CAD) and cardiovascular death were higher in prolonged P-R interval groups. The chi-square test showed this difference was statistically non-significant (*p* > 0.05). The multivariate logistic regression model revealed non-significant relationships between prolonged P-R interval and SYNTAX score, significant CAD, three-vessel disease, inverted T wave, anterior ST depression, heart rate and HDL.

**Conclusions:**

Based on the results of our study the six-month follow-up showed non-significant outcomes. Further studies are recommended to assess the long-term outcomes.

## Background

P-R interval is defined as the beginning time of the atrial depolarization to the onset of ventricular depolarization and on the electrocardiography (ECG) measured from the P wave to the beginning of the QRS complex. The P-R interval of more than 200 milliseconds is considered prolonged on the ECG [1]. Prolonged P-R interval might have a positive correlation with cardiovascular outcome [2]. The results of a study showed in the case of coronary artery disease (CAD) prolonged P-R interval is associated with adverse clinical outcomes including hospitalization following heart failure and death [3]. Another study revealed prolonged P-R interval increased the risk of atrial fibrillation and also pacemaker implantation [4]. The results of a Japanese study showed P-R interval of more than 200 milliseconds was a predictor of the development of atrial tachyarrhythmias in patients who underwent tetralogy Fallot repair [5]. Nevertheless, the role of prolonged P-R interval in CAD is unclear [3]. Based on the results of an Iranian study, a prolonged PR interval is independently associated with significant CAD in patients with acute coronary syndrome. So that, patients with prolonged PR intervals were statistically associated with significant CAD. Also, they had a higher trend Gensini scores and a higher frequency of left main coronary artery or three‑vessel CAD [6]. Another study assessed 915 ST elevation myocardial infarction (STEMI) patients. The results showed prolonged P-R interval was an independent predictor of long-term mortality [7]. However, the results of another study revealed P-R interval of more than 190 milliseconds was significantly associated with a higher incidence of atrial fibrillation, heart failure and death in patients with implanted cardiac devices [8].

Based on the results of a study, prolonged PR was correlated with endothelial dysfunction and increased pulse-wave velocity even in healthy participants [9]. Endothelial dysfunction indicates global functional dysfunction of the cardiovascular system. Prolonged PR interval could occur as a process of aging and degeneration of the conduction system. Also, atherosclerosis is correlated with aging. So, the prolonged PR interval could serve as a clinical marker for cardiovascular events [10]. So, the role of prolonged PR interval is closely related to cardiovascular pathological entities including myocardial infarction (MI), ischemic stroke, and congestive heart failure (CHF) [11–13]. Based on the result of a Chinese study P-R interval of more than 200 milliseconds was significantly associated with higher carotid intima media thickness. Also, the result of the last study showed prolonged P-R interval was an independent predictor of ischemic stroke and cardiovascular death [10]. However, the results of a study in the general population showed a non-significant relationship between prolonged P-R interval and heart failure, stroke, hospitalization secondary to CAD, atrial fibrillation and mortality [14].

The long-term clinical outcome and prognostic significance of prolonged PR interval have not been assessed in patients who were hospitalized with NSTEACS including unstable angina or NSTEMI. Also, some clinical outcomes were controversial. Hence, we investigated the present study to respond to clinical question, can prolonged P-R interval predict clinical outcomes in non-ST elevation acute coronary syndrome patients?

## Methods

This descriptive analytical study was conducted on NSTEACS patients referred to the emergency department of Mazandaran Heart Center, Sari, Iran from April 2019 to July 2020. This center is affiliated with Mazandaran University of Medical Sciences and is the only specialized heart center in Mazandaran province.

### Sampling

All of the NSTEACS patients were selected via convenience sampling method and then divided into two groups: prolonged P-R interval (P-R interval > 200 msec) and normal P-R interval (120–200 msec) based on the ECG in lead II. The patients who had a history of digoxin and calcium channel blocker use, using antiarrhythmic drugs, known valvular or congenital heart disease and connective tissue, unreadable P-R interval and cardiac block were excluded. The sample size was calculated by 126 participants in each group according to the results of the Aro et al. study (σ^2^ = 289) and d = 3, α = 0.05 [14].

### Definition of NSTEACS patients

The NSTEACS patients were diagnosed by clinical manifestation of the coronary syndrome, ECG change (Inverted T-wave and ST segment depression ≥ 0.5 mm) and the level of cardiac biomarkers [15].

### ECG data

All NSTEACS underwent standard 12-lead ECG with the ECG paper speed of 25 mm/s and voltage 10 mm/mv [16] within 30 min of emergency department arrival. Then two independent cardiologists who were blinded to the study determined the P-R interval on ECGs. Also, the interobserver correlation coefficient was 0.91 to assess the reliability of the P-R interval calculation by 2 independent evaluators in ten randomly selected patients.

### Echocardiography data

Transthoracic echocardiography was performed by a cardiologist for all NSTEACS patients after admission by Vivid S5 (GE Healthcare, Wauwatosa, WI, USA) with a 1–3 MHz transducer. Left ventricular ejection fraction (LVEF) was calculated as the end-diastolic volume (EDV) minus the end-systolic volume divided by the EDV from biplane apical two-chamber and four-chamber views using a modified Simpson’s technique [17].

### Coronary angiography data

All patients underwent coronary angiography by Siemens AG, Medical Solutions, Erlangen, Germany, 48 to 72 h after admission. One cardiologist who was blinded to the study reported all angiograms. Significant CAD was defined as at least 50% stenosis in the left main coronary artery or at least one coronary artery stenosis of more than 70% [18]. Gensini score, widely used angiographic scoring system, was measured to assess the severity of atherosclerosis and consider 3 main parameters for each coronary lesion: severity score, region multiplying factor and collateral adjustment factor [19]. A SYNTAX score was used to define the coronary atherosclerotic lesion. Similar to the Gensini score, the SYNTAX score was calculated by two cardiologists who were blinded in the study. SYNTAX score of more than 20 is defined as high SYNTAX score [20].

### Data collection tool

The questionnaire had been completed by one of the researchers. It consisted of three sections. First, the demographic data including age, gender and medical history, had been obtained. In the second part clinical outcomes including the results of transthoracic echocardiography (left ventricular ejection fraction, mitral valve insufficiency and left ventricular diastolic dysfunction) and CAG (Left main coronary artery stenosis at least 50% and more, another main coronary artery stenosis at least 70% and more, Gensini score) were assessed. After six months, the third follow-up part including mortality, ACS and cerebrovascular accident had been completed.

### Data analysis

Data analysis was performed using the SPSS software version 21 (version 24; Chicago, IL). The mean ± standard deviation was used to describe the age, LVEF, number of leads with STD more than 0.1mv, Gensini score, the levels of HDL, LDL, TG, Chol, FBS, troponin, CKMB and heart rate. Also, the frequency and percentage were utilized to describe the gender, hypertension, hyperlipoproteinemia, positive family history, etc. To assess the difference between the two groups (normal P-R interval and prolonged P-R interval) in terms of age, heart rate, LVEF, Gensini score, number of leads with STD more than 0.1mv, biochemistry laboratory tests the Mann-Whitney u test was used. Also, Fisher exact test and chi-square were utilized to compare the two groups (normal P-R interval and prolonged P-R interval) in terms of gender, hypertension, hyperlipoproteinemia, positive family history, mitral regurgitation, diastolic dysfunction, CAD, cardiovascular death, ischemic stroke, etc. Univariate and multivariate logistic regression models were subsequently performed to explore potential predictors for prolonged P-R intervals. Odds ratio (OR) and 95% CI were similarly obtained from the logistic regression models. Variables with a significance level of less than 0.2 were included in the multivariate regression model. Furthermore, *p* value less than 0.05 was considered statistically significant.

## Results

According to Table 1, both of the two groups, normal P-R interval and prolonged P-R interval, had non-significant differences in terms of age (*p* = 0.563), gender (*p* = 0.254), history of diabetes mellitus (*p* = 0.290), hypertension (*p* = 0.187), hyperlipoproteinemia (*p* = 0.905), positive family history (*p* = 0.770) and previous CABG (*p* = 0.429). However, the results of the chi-square test showed a statistically significant difference between the two groups in terms of the history of MI (*p* = 0.018).

**Table 1:** Demographic and medical characteristics of patients stratified by P-R interval time

Variable		Normal P-R interval (*N* = 142)	Prolonged P-R interval (*N* = 106)	Statistical test & *p*-value
Age (Year)		58.17 ± 9/73	59.08 ± 11.65	0.563*
Gender	Male	70 (49.3)	60 (57.6)	0.254**
	Female	72 (50.7)	46 (43.4)	
DM		52 (36.6)	32 (30.2)	0.290**
HTN		79 (55.6)	50 (47.2)	0.187**
HLP		49 (34.5)	37 (34.9)	0.905**
Positive FH		12 (8.5)	10 (9.5)	0.770**
History of MI		27 (19.0)	34 (32.1)	0.018**
History of CABG		4 (2.8)	5 (4.7)	0.429**

* Mann-Whitney u test **Chi square

Based on the Table 2, laboratory indices had non-significant differences in terms of the level of LDL (*p* = 0.596). TG (*p* = 0.935), cholesterol (*p* = 0.666), FBS (*p* = 0.823), troponin (*p* = 0.963) and CK-MB (*p* = 0.968). But there was a significant difference between the two groups in terms of the level of HDL (*p* = 0.004).

**Table 2:** Laboratory finding of patients stratified by P-R interval time

Variable	Normal P-R interval	Prolonged P-R interval	Statistical test & *p*-value
HDL	39.69 ± 10.58	36.75 ± 11.88	0.004*
LDL	109.26 ± 32.68	106.84 ± 37.22	0.596*
TG	211.11 ± 129.61	212.25 ± 126.87	0.935*
Chol	181.70 ± 46.31	184.48 ± 53.86	0.666*
FBS	126.11 ± 73.42	128.23 ± 77.66	0.823*
Troponin	4.63 ± 14.36	4.40 ± 19.65	0.963*
CK-MB	44.53 ± 65.06	37.61 ± 36.56	0.968*

* Mann-Whitney u test

The cardiac para-clinical findings of patients stratified by P-R interval time is presented in Table 3. The finding of the ECG showed patients had significant differences in terms of the heart rate (*p* = 0.042), inverted T wave (*p* = 0.017), anterior ST- Segment depression (*p* = 0.008) and SYNTAX score (*p* = 0.032). So, the patients with prolonged P-R interval had lower heart rates, high frequency of anterior ST- segment depression and higher mean of SYNTAX score but higher frequency of inverted T-wave was in the normal P-R interval group.

**Table 3:** Cardiac para-clinical finding of patients stratified by P-R interval time

Variable			Normal P-R interval (*N* = 142)	Prolonged P-R interval (*N* = 106)	Statistical test & *p*-value
ECG	HR (BPM)		75.11 ± 15.03	71.08 ± 14.40	0.042*
	RBBB		5 (3.5)	2 (1.9)	0.702**
	ST depression ≥ 0.1mv		94 (66.2)	75 (70.8)	0.446**
	Number of lead with STD ≥ 0.1mv		2.30 ± 2.64	2.87 ± 2.62	0.084*
	Non ischemic change		40 (28.2)	29 (27.5)	0.924**
	Inverted T wave		33 (23.2)	12 (11.5)	0.017**
	Inferior STD		7 (5.0)	3 (2.5)	0.406**
	Lateral STD		4 (2.8)	1 (0.9)	0.396**
	Anterior STD		41 (28.8)	48 (45.4)	0.008*
	Inferolateral STD		3 (2.1)	3 (2.5)	0.716**
	Antrolateral STD		12 (8.5)	10 (9.7)	0.788**
	Inferoanterolateral STD		2 (1.4)	0 (0.0)	0.509***
Echocardiography	LVEF		50.43 ± 7.36	50.63 ± 7.65	0.605*
	Diastolic dysfunction (*N* = 241)	No	75 (54.3)	56 (54.4)	0.479***
		Grade I	61 (44.2)	44 (42.7)	
		Grade II	1 (0.7)	3 (2.9)	
		Grade III	1 (0.7)	0 (0.0)	
	MR (*N* = 239)	No	48 (35.0)	34 (33.3)	0.469**
		Mild	63 (46.0)	48 (47.1)	
		Moderate	23 (16.8)	14 (13.7)	
		Severe	3 (2.2)	6 (5.9)	
CAG (*N* = 246)	Normal CAG		19 (13.6)	3 (2.8)	0.003**
	SVD		19 (13.6)	18 (17.0)	0.459**
	2VD		44 (31.4)	28 (26.4)	0.392**
	3VD		36 (25.7)	40 (37.7)	0.043**
	MVD		6 (4.3)	3 (2.8)	0.547**
	Left main lesion		0 (0.0)	3 (2.8)	0.045***
	Minimal CAD		16 (11.4)	11 (10.4)	0.794**
	Gensini score		41.5 ± 34.30	44.32 ± 44.69	0.087*
	SYNTAX score		35.90 ± 45.15	44.01 ± 43.24	0.032*

* Mann-Whitney u test **Chi square ***Fisher exact test

Echocardiography findings of the patients revealed non-significant difference in both groups, normal P-R interval and prolonged P-R interval, in terms of ejection fraction, diastolic dysfunction and mitral regurgitation (*p* > 0.05).

The reports of the coronary angiography revealed the frequency of normal CAG was higher in the normal P-R interval group compared to the prolonged P-R interval group (*p* = 0.003). Also, the frequency of three vessels disease (*p* = 0.043) and left main lesion (*p* = 0.045) was higher in the prolonged P-R interval group compared to the normal P-R interval group.

The results of the six-month follow-up are provided in Table 4. Although, the frequency of ischemic stroke, CAD and cardiovascular death were higher in prolonged P-R interval groups but the chi-square test showed these differences were statistically non-significant (*p* > 0.05).

**Table 4:** Six months follow up of patients stratified by P-R interval time

Variable	Normal P-R interval	Prolonged P-R interval	Statistical test & *p*-value
Ischemic stroke	1 (0.7)	3 (2.9)	0.318*
CAD	1 (0.7)	2 (1.9)	0.579*
Cardiovascular death	2 (1.5)	3 (3.0)	0.655*

* Fisher exact test

Table 5 shows the predictors of the P-R interval. The univariate logistic regression model revealed significant relationships between prolonged P-R interval and significant CAD (OR: 0.457, 95% CI: 0.231–0.901, *p* = 0.024), three vessel disease (OR: 0.571, 95% CI: 0.331–0.986, *p* = 0.044), inverted T wave (OR: 0.422, 95% CI:0.206–0.863, *p* = 0.018), anterior ST depression (OR: 2.039, 95% CI: 1.203–3.454, *p* = 0.008).

**Table 5:** Logistic regression for predictors of prolonged P-R interval

Variables		Univariate			Multivariate		
		OR	95% CI	*p*-value	OR	95% CI	*p*-value
SYNTAX score	≥ 20	1.458	0.871–2.440	0.152	1.130	0.538–2.375	0.747
	< 20	Ref	Ref	Ref	Ref	Ref	Ref
Significant CAD	Yes	0.457	0.231–0.901	0.024	1.886	0.789–4.510	0.154
	No	Ref	Ref	Ref	Ref	Ref	Ref
3VD	Yes	0.571	0.331–0.986	0.044	1.123	0.532–2.245	0.742
	No	Ref	Ref	Ref	Ref	Ref	Ref
Inverted T wave	Yes	0.422	0.206–0.863	0.018	0.524	0.217–1.268	0.152
	No	Ref	Ref	Ref	Ref	Ref	Ref
Anterior STD	Yes	2.039	1.203–3.454	0.008	1.510	0.799–2.853	0.204
	No	Ref	Ref	Ref	Ref	Ref	Ref
Heart rate (bpm)	≤ 60	1.399	0.806–2.429	0.189	1.505	0.810–2.796	0.196
	> 60	Ref	Ref	Ref	Ref	Ref	Ref
HDL (mg/dl)	≤ 40	1.674	0.799–3.508	0.172	1.955	0.812–4.707	0.135
	> 40	Ref	Ref	Ref	Ref	Ref	Ref

The multivariate logistic regression model revealed non-significant relationships between prolonged P-R interval and SYNTAX score (OR: 1.130, 95% CI: 0.538–2.375, *p* = 0.747), significant CAD (OR:1.886, 95% CI: 0.789–4.510, *p* = 0.154), three-vessel disease (OR: 1.123, 95% CI:0.532–2.245, *p* = 0.742), inverted T wave (OR: 0.524, 95% CI: 0.217–1.268, *p* = 0.152), anterior ST depression (OR: 1.510, 95% CI: 0.799–2.853, *p* = 0.204), heart rate (OR: 1.505, 95% CI: 0.810–2.796, *p* = 0.196) and HDL (OR: 1.955, 95% CI: 0.812–4.707, *p* = 0.135).

## Discussion

The present study aimed to respond to clinical question, can prolonged P-R interval predict clinical outcomes in non-ST elevation acute coronary syndrome patients? To address this clinical challenge, we used comprehensive assessment including demographic, laboratory indices, electrocardiography, echocardiography, coronary angiography and also six-month follow-up. In the present study, 32.1% of patients in the prolonged P-R interval group had previous MI and this frequency was statistically significant compared to the normal P-R interval group. This result was confirmed by other studies. In a study conducted in Pakistan, the results showed 8.7% of acute MI patients had high degree AV block [21]. This prevalence was 13.9% in a study [22]. Another study in Pakistan revealed that14% of inferior ST-elevation MI patients had persistent AV node block [23].

In our study, the HDL level was significantly lower in the prolonged P-R interval group. Similar to our reports, the results of the Chinese study showed low HDL level was associated with an increased risk of AV node block in patients who were referred for health routine assessment [24]. This may be due to the protective characteristics of HDL against inflammation and oxidative stress in endothelial cells [25].

In the present study, the mean heart rate was significantly lower in prolonged P-R interval groups. In this line, the results of a study showed the risk of AV node block was positively associated with lower heart rate [24]. In most cases, the lower heart rate was accompanied by types of the AV block [26].

Our study revealed significantly high frequency of anterior ST depression in prolonged P-R interval groups. ST-segment depression indicates ischemia following conduction abnormalities [27]. Although, the inferior leads change may have been associated with the prolonged P-R interval. This finding in our study may be due to the reciprocal pattern [28].

In the current study, the significant high frequency of normal coronary and three-vessels disease was observed in normal P-R interval groups and prolonged P-R interval groups, respectively. Based on the Pakistani study which assessed patients with heart block, 17.9% of ischemic heart disease patients had three- vessel disease [29]. This is due to hypoperfusion of the AV nodal artery that is mainly supplied by the right coronary artery and some cases from a left circumflex artery [30]. So, AV blocks may accompany right coronary artery occlusion [31]. Also, the results of a narrative study showed that patients with inferior MI and left anterior descending artery obstruction may have a significantly higher risk of developing complete AV node block [32].

In the current study, despite the high frequency of cardiovascular death in the prolonged P-R interval group, this difference was non-significant. The results of a study in Serbia showed patients with complete heart block had higher significant long-term mortality rates compared to others [33]. This difference may be due to the time of follow-up. In the last, study 6-year follow-up was done. Also, the prevalence of the ischemic stroke event was not significantly higher in the prolonged P-R interval group. While in an Iranian cohort study, the results showed the prevalence of AV block was significantly higher in patients who died following ischemic stroke [34].

Based on the results of our study, some variables including patients with a history of previous MI, lower heart rate, lower HDL level, anterior ST-depression, inverted T wave, lower normal coronary, higher frequency of three-vessel disease and multi-vessel disease could experience prolonged P-R interval. But the six-month follow-up showed non-significant outcomes. The limitation of the present study was the data of the single center. The long- period cohort study will be beneficial in assessing the long-term outcomes. Also, it is recommended to find a relation between the culprit arteries in patients with prolonged PR interval.

## Data Availability

Due to the privacy of the research participants, the data of the present study are available from the corresponding author upon reasonable request.

## Abbreviations

NSTEACS: Non ST elevation acute coronary syndrome
ECG: Electrocardiography
CAD: Coronary Artery Disease
NSTEMI: Non‑ST‑segment elevation myocardial infarction
CAG: Coronary Angiography
DM: Diabetes Mellitus
HTN: Hypertension
HLP: Hyperlipoproteinemia
FH: Family history
MI: Myocardial Infarction
CABG: Coronary Artery Bypass Graft
STD: ST segment depression
HDL: High Density Lipoprotein
LDL: Low Density Lipoprotein
TG: Triglyceride
Chol: Cholesterol-
FBS: Fasting Blood Sugar
CK-MB: Creatine Kinase-Myoglobin Binding
HR: Heart Rate
RBBB: Right Bundle Branch Block
LVEF: Left Ventricle Ejection Fraction
MR: Mitral Regurgitation
CAD: Coronary Artery Disease
SVD: Single Vessel Disease
BPM: Beat Per Minute
